# Characterization of Defatted Products Obtained from the Parmigiano–Reggiano Manufacturing Chain: Determination of Peptides and Amino Acids Content and Study of the Digestibility and Bioactive Properties

**DOI:** 10.3390/foods9030310

**Published:** 2020-03-09

**Authors:** Sofie Buhler, Ylenia Riciputi, Giuseppe Perretti, Maria Fiorenza Caboni, Arnaldo Dossena, Stefano Sforza, Tullia Tedeschi

**Affiliations:** 1 Department of Food and Drug, University of Parma, Parco Area delle Scienze 27/A, I-43124 Parma, Italy; sofie.buhler@yahoo.it (S.B.); arnaldo.dossena@unipr.it (A.D.); stefano.sforza@unipr.it (S.S.); 2 Department of Agro-Food Sciences and Technologies, Alma Mater Studiorum-University of Bologna, Piazza Goidanich 60, I-47521 Cesena (FC), Italy; ylenia.riciputi@yahoo.it (Y.R.); maria.caboni@unibo.it (M.F.C.); 3 Department of Agricultural, Food and Environmental Sciences, University of Perugia, Via S. Costanzo, n.c.n., 06126 Perugia, Italy; giuseppe.perretti@unipg.it

**Keywords:** defatted cheese, peptides, amino acids, bioactivity, digestibility

## Abstract

Parmigiano–Reggiano (PR) is a worldwide known Italian, long ripened, hard cheese. Its inclusion in the list of cheeses bearing the protected designation of origin (PDO, EU regulation 510/2006) poses restrictions to its geographic area of production and its technological characteristics. To innovate the Parmigiano–Reggiano (PR) cheese manufacturing chain from the health and nutritional point of view, the output of defatted PR is addressed. Two defatting procedures (Soxhlet, and supercritical CO_2_ extraction) were tested, and the obtained products were compared in the composition of their nitrogen fraction, responsible for their nutritional, organoleptic, and bioactive functions. Free amino acids were quantified, and other nitrogen compounds (peptides, proteins, and non-proteolytic aminoacyl derivatives) were identified in the extracts and the mixtures obtained after simulated gastrointestinal digestion. Moreover, antioxidant and angiotensin converting enzyme (ACE) inhibition capacities of the digests were tested. Results obtained from the molecular and biofunctional characterization of the nitrogen fraction, show that both the defatted products keep the same nutritional properties of the whole cheese.

## 1. Introduction

Parmigiano–Reggiano (PR) is a worldwide known Italian hard-cooked and slowly-matured cheese. Its inclusion in the list of cheeses bearing the protected designation of origin (PDO, EU regulation 510/2006) poses restrictions to its geographic area of production and its technological characteristics. Parmigiano Reggiano P.D.O. is made from raw cow’s milk, partially skimmed by natural surface skimming and produced by cows whose feed consists mainly of forage grown in the area of origin. The milk may not undergo any heat treatment, and no additives may be used [1]. It is a highly concentrated cheese and contains only 30% water and 70% nutrients: 30% protein, 30% fat. The remaining 10% is composed of vitamins, mineral salts, and free amino acids. Free amino acid fraction, which generally increases during cheese maturation, gives an essential contribution to the original taste of the product [2]. The fat content of dairy products is a crucial feature since many studies have been reported concerning their possible adverse effects on human health. Cow milk is, in fact, rich in saturated fatty acids (approximately 70% of total milk fat [3], the intake of which has been associated with an elevated risk of cardiovascular disease (CVD) [4] through increased plasma cholesterol and low-density lipoprotein (LDL) [5]. Moreover, the increased consumption of dietary fat in industrialized nations has been related to some types of cancer and obesity [6]. For those reasons, consumer demand for low-fat food products is steadily growing [7,8].

Lowering fat in cheese manufacturing can be quite easily performed by using totally skimmed milk for production. Still, it is commonly reported that low-fat cheese often suffers from undesirable flavor and texture [9]. Therefore, different approaches have been developed, such as processing techniques, adjunct cultures, use of additives such as fat replacers, and fat removing methods. It has been reported by Whetstine et al. [10] that the flavor release is different in the mouth with reduced-fat products than in full-fat products because hydrophobic flavor molecules have a higher sensory threshold in oil than they do in the water. When fats are extracted from milk before the cheese is made, there are fewer fat molecules for the sensory compounds to bind to, resulting in a lack of flavor reduced-fat cheese. Developing an extract method for defatting cheese is also useful to keep the same manufacturing chain of the new cheeses, following indications for the PDO rules. The use of supercritical fluid extraction technology for the production of low-fat parmesan and cheddar cheese has been previously reported [11].

The objective of this work is the complete characterization of the nitrogen fraction of two samples of defatted Parmigiano–Reggiano obtained with CO_2_ supercritical extraction technology and Soxhlet procedure. In particular, the nutritional content of the products was evaluated in terms of peptide and amino acid content. Moreover, digestibility, antioxidant, and ACE-inhibitor properties were tested in vitro.

## 2. Materials and Methods

### 2.1. Cheese Defatting

A cheese sample aged 24 months was obtained from the Parmigiano–Reggiano Consortium (Reggio Emilia, Italy). Defatting was performed according to the following procedures. Soxhlet extraction procedure: The fat was extracted from 10 g of grated cheese, according to a slightly modified method of Manirakiza et al. [12], with 60 mL hexane for 2 h in a Soxhlet apparatus. Each extraction was performed in duplicate. Supercritical CO_2_ extraction procedure: The SFE (Spe-ed SFETM; Applied Separation Allentown, PA, USA) conditions for total fat removal, according to a slightly modified method of Perretti et al. [13], were 24 g (50-mL vessel) grated cheese; pressure: 60 MPa; extractor temperature: 353 K; CO_2_ flow rate: 0.990 g/L; static extraction time: 5 min; dynamic extraction time: 60 min. The extraction was performed five times.

### 2.2. Fatty Acids Determination by GasChromatrography( GC-FID)

The fatty acids (FA) composition of Parmigiano–Reggiano cheese was determined from the lipid fraction as FAMEs by capillary gas chromatography analysis, as reported by Verardo et al. [14].

To convert fatty acids to their corresponding methyl esters(FAMEs), the method of Christie [15] was used. The phospholipid fraction obtained from TLC separation was transmethylated and the FAMEs were analyzed by capillary gas chromatography using a BPX70 fused silica capillary column (10 m × 0.1 mm i.d., 0.2 μm film thickness; SGE Analytical Science, Ringwood, VIC, Australia). The column was fitted on a GC-2010 Plus gas chromatograph (Shimadzu, Tokyo, Japan). The injector and flame ionization detector temperatures were set at 240 °C. Hydrogen was used as carrier gas at a flow rate of 0.8 mL min^−1^. The oven temperature was held at 50 °C for 0.2 min, increased from 50 to 175 °C at 120 °C min^−1^, held at 175 °C for 2 min, increased from 175 to 220 °C at 20 °C min^−1^, and finally risen from 220 to 250 °C at 50 °C min^−1^. Samples were injected in split mode (0.4 μL) with a split ratio set at 1:10. Peak identification was accomplished by comparing peak retention times with GLC-463 and FAME 189-19 standard mixtures.

### 2.3. Cholesterol Analysis by GC-FID

The lipid fraction was saponified at room temperature using 10 mL of methanolic 0.5 M KOH for 18 h in the dark under constant stirring. After saponification, the organic fraction was washed with deionized water, and the unsaponifiable matter was extracted three times with diethyl ether. The organic fractions were pooled together and the solvent was removed under vacuum. The unsaponifiable matter was stored in *n*-hexane/2-propanol (4/1 *v/v*) at −18 °C until GC analysis. GC analyzed the previous extract after silylation. The trimethylsilyl derivatives (TMS) of sterols were analyzed as reported by Guerra et al. [16] by GC-FID. The analysis was carried out in duplicate for each sample.

### 2.4. Total Nitrogen and Fat Content Determination

For total nitrogen determination, and consequently, the determination of the protein content, the Kjeldahl instrument was used following the standard protocol, according to the European Regulation EC 152/20096. Analyses were performed in duplicate on the whole cheese and on the two defatted products.

The fat content determination was performed by using the Soxhlet method following the standard protocol, according to AOAC Official Method 948.22. [17] Analyses were performed in duplicate on the control sample and on the two defatted products.

### 2.5. Isolation of the Peptide and Amino Acid Fractions 

The defatted samples, in an amount corresponding to 1 g of the treated cheese, were suspended in 4.5 mL of HCl 0.1 M; the dipeptide Phe-Phe was added as an internal standard to a final concentration of 50 µM. Control samples were prepared in the same way using 1 g of untreated cheese. The produced suspensions were homogenized by Ultraturrax (90”, 14,000 rpm) and centrifuged for 30′ at 5 °C; the samples were then filtered on 0.45 µm membranes to obtain clear extracts that were analyzed directly by LC-MS. All samples were prepared in triplicate.

### 2.6. Simulated Gastrointestinal Digestion

The defatted cheese samples and a whole cheese sample were digested according to the procedure described by Minekus et al. [18], starting with an amount of defatted sample corresponding to 1 g of untreated cheese. Briefly, 1 mL of salivary buffer (12.08 mM KCl, 2.96 mM KH_2_PO_4_, 10.88 mM NaHCO_3_, 0.12 mM MgCl_2_, and 0.048 mM (NH4)_2_CO_3_, 0.6 mM CaCl_2_), containing 75 U/mL amylase, was added to an amount of sample corresponding to 1 g of untreated cheese. The sample was vortexed and incubated for 2 min at 37 °C. Then, 2 mL of gastric buffer (5.52 mM KCl, 0.72 mM KH_2_PO_4_, 20 mM NaHCO_3_, 37.76 mM NaCl, 0.08 mM MgCl_2_, 0.4 mM (NH4)_2_CO_3_ and 0.06 mM CaCl_2_), containing 6250 U/mL pepsin, adjusted to pH = 3 with HCl were added. The mixture was vortexed and incubated for 2 h at 37 °C. Finally, 4 mL of intestinal buffer (5.44 mM KCl, 0.64 mM KH_2_PO_4_, 68 mM NaHCO_3_, 30.72 mM NaCl, 0.264 mM MgCl_2_ and 0.24 mM CaCl_2_), containing 100 U/mL pancreatin and 37.5 mg/mL bile, adjusted to pH = 7 with NaOH, were added (final ratio cheese:digestive fluids 1:7, *w:v*). The sample was vortexed and incubated for 2 h at 37 °C. The digestion was stopped heating the sample at 95 °C for 15 min. After cooling, 125 the samples were centrifuged for 45 min at 4 °C at 3220× *g*.

A digestion blank was obtained by treating 1 mL of H_2_O to the same procedure. Each sample was prepared in triplicate. Samples for LC–MS analyses were prepared by adding Phe-Phe as an internal standard to a final concentration of 50 µM, to the clear supernatants.

### 2.7. LC-MS Amino Acids Quantification

First, 50 µL of the previously obtained extracts or the clear supernatants produced after the simulated digestion process were mixed with 50 µl of 2.5 mM norleucine in 0.1 M HCl; 10 µL of the produced solutions were derivatized with the Waters AccQ-Fluor reagent kit, according to the instructions of the manufacturer. The derivatized samples were analyzed on a UPLC/ESI–MS system (UPLC Acquity Waters with a single quadrupole mass spectrometer Waters Acquity Ultra performance, Waters, Milford, MA, USA) using a RP column (ACQUITY UPLC BEH 300 C18 1.7 µm 2.1 × 150 mm, Waters, Milford, MA, USA) and a gradient elution. Eluent A was H_2_O with 0.1% formic acid, eluent B was acetonitrile with 0.1% formic acid; gradient: 0–7 min 100% A, 7–30 min from 100% A to 73.3% A; flow: 0.2 mL/min; column temperature: 35 °C; sample temperature: 18 °C; injection volume: 2 μL. The samples were analyzed in the SIR Scan mode (monitored ions are reported in Table 1); ionization type: positive ions; capillary voltage: 3.2 kV; cone voltage: 30 V; source temperature: 150 °C; desolvation temperature: 300 °C; cone gas flow: 100 l/h; desolvation gas flow: 650 l/h. 

Calibration curves for the amino acids were obtained as follows: amino acid standard H solution (Thermo Scientific, Rockford, IL, USA) was mixed with an equal volume of norleucine 2.5 mM in HCl 0.1 M; 1:2, 1:4, 1:8, and 1:16 dilutions in H_2_O were prepared, and the produced solutions were derivatized and analyzed as previously mentioned for the samples. The calibration curve for tryptophan, asparagine, and glutamine was obtained in the same way, after mixing equal volumes of a solution containing these three amino acids at a concentration of 2.5 mM each, in HCl 0.1 M and a solution containing norleucine 2.5 mM in HCl 0.1 M. In the case of the digests, also the blank digestion was analyzed according to the procedure outlined above. The produced results were subtracted from the values obtained for the digested cheese samples.

### 2.8. LC–MS Characterization of Peptides and Proteins

The extracts and the digested samples were analyzed on a UPLC/ESI–MS system (UPLC Acquity Waters with a single quadrupole mass spectrometer Waters Acquity Ultraperformance, Waters, Milford, MA, USA) using a RP column (ACQUITY UPLC BEH 300 C18 1.7 µm 2.1 × 150 mm, Waters, Milford, MA, USA) and a gradient elution. Eluent A was H_2_O with 0.1% formic acid, eluent B was acetonitrile with 0.1% formic acid; gradient: 0–7 min 100% A, 7–47 min from 100% A to 53.5% A; flow: 0.2 mL/min; column temperature: 35 °C; sample temperature: 18 °C; injection volume: 6 μL. The samples were analyzed in the Full Scan mode; ionization type: positive ions; scan range: 100–2000 m/z; capillary voltage: 3.2 kV; cone voltage: 30 V; source temperature: 150 °C; desolvation temperature: 300 °C; cone gas flow: 100 l/h; desolvation gas flow: 650 l/h. Each produced chromatogram was elaborated, determining characteristic ions, molecular weights, possible in-source collision-induced dissociation (CID) fragments of the main peaks and retention times. In the case of molecular weights higher than 2000 Da, the value was confirmed by the MaxEnt application of MassLynx Software (Waters, Milford, MA, USA). The integration of the area of each compound was performed by QuanLynx software (Waters, Milford, MA, USA), after the extraction of the characteristic XICs. The integrated areas of each species in each extract were determined, and every compound was semi-quantified by dividing by the area of the internal standard (Phe-Phe) in the same sample (Semi-quantification value = Area compound/Area Phe-Phe).

### 2.9. Antioxidant Capacity of the Digested Samples

The antioxidant capacity was measured by ABTS assay, according to the method proposed by Re et al. [19] with slight modifications. ABTS (2,20-azinobis(3-ethylbenzothiazoline-6-sulfonic acid) and potassium persulfate (K_2_S_2_O_8_) were dissolved in H_2_O to obtain concentrations of 70 and 2 mM, respectively. The stock solution containing the ABTS radical cation was generated by adding 1% (*v/v*) of K_2_S_2_O8 to the ABTS solution and incubating the mixture overnight in the dark. The ABTS working solution was prepared by diluting the ABTS stock solution 1:25 in phosphate buffer solution (PBS; pH = 7.4), to obtain an absorbance of about 0.7 ± 0.02. The digested samples and the digestion blank were diluted 1:500, and 200 µL of the obtained solutions were added to 1.8 mL of ABTS working solution. The control solution was made by mixing 0.2 mL of PBS with 1.8 mL of work solution. All the samples were incubated for 1 h in the dark before the measurement of the absorbance at 734 nm, performed using a Jasco V-530 UV–vis Spectrophotometer (Jasco Inc, Easton, USA). To express the antioxidant capacity in terms of TEAC (Trolox equivalent antioxidant capacity—mmol of Trolox for mL of digested sample), aqueous solutions containing variable amounts of Trolox were analyzed with the same procedure outlined for the samples. All samples and standards were analyzed in duplicate.

### 2.10. ACE Inhibition Capacity of the Digested Samples

The percentage of ACE inhibitory activity for the digested samples was determined using the methods of Cushman et al. and Nakamura et al. [20,21] with slight modifications. The following solutions were prepared: sodium borate buffer (0.1 M, NaBB) with NaCl (300 mM), pH 8.3; potassium phosphate buffer (0.01 M, KPB) with NaCl (500 mM), pH 7; 5 mM hippuryl-histidyl-leucine (HHL) in NaBB buffer; and ACE 0.1 U/mL in KPB + 5% glycerol (g/mL). The following samples were prepared: ACEmax = 100 μL of HHL + 40 μL of NaBB + 10 μL of ACE; ACEmin = 100 μL of HHL + 40 μL of digested sample (diluted 1:100) + 10 μL of ACE. After 60 min of incubation at 37 °C, the reaction was quenched adding 125 μL of HCl 1 M. The analysis was performed by HPLC–UV (Alliance 2695 separation, Waters, Milford, MA, USA) with dual λ absorbance detector model 2487 (Waters), using an RP JUPITER C18 column (5 μm, 300 Å, 250 × 2 mm, Phenomenex, Torrance, CA, USA); eluent A: H2O with 0.1% trifluoroacetic acid; eluent B: acetonitrile with 0.1% trifluoroacetic acid; gradient elution: 0–10 min, 100% A; 10–25, linear from 100 to 33% A; flow, 0.2 mL/min; column temperature, 35 °C; injection volume: 10 μL; UV detection, λ = 228. Data analysis was performed with Empower software (Waters, Milford, MA, USA). The integration values for the peak eluting at 22.5’, corresponding to hippuric acid (HA), were used to calculate the inhibition values, according to the following equation: I% = (Area HA(ACEmax) − Area HA (ACEmin))/Area HA (ACEmax) × 100.

### 2.11. Data Analysis

Statistical analyses were performed using the Statistical Package for the Social Sciences (SPSS for Windows version 11.0; SPSS Inc., Chicago, IL, USA). One-way analyses of variance (ANOVA) were run using Duncan’s test.

## 3. Results and Discussion

### 3.1. Defatted Products

PR aged 24 months, which is the most consumed aging, was obtained from the Parmigiano–Reggiano Consortium and defatted according to 2 different procedures: Soxhlet and supercritical CO_2_ extraction. The obtained yields in the defatted product were comparable, respectively: 65% and 66%. 

Defatted products were analyzed for their total nitrogen and fat content, as reported in Table 1.

As shown in Table 1, the total protein amount is higher in the two defatted products, due to an effect of concentration. Considering the residual fat content, both the methods are very efficient if compared with the literature [10,11], since the reduction of fat far exceeds 50%. 

At the same time, the quality of the fat extracted with the two extraction methods was assessed by fatty acids gas chromatographic evaluation. The composition did not show significant differences between the two extracts: the primary fatty acid was palmitic acid (28.6%) and oleic acid (22.8%); in both cases, cholesterol represented 2.7% of the fat. This content was comparable to what declared from the nutritional composition by Parmigiano–Reggiano Consortium.

Thus, these results confirm that the methods chosen for lipid extraction were able to recover, efficiently, both total fatty acids and cholesterol.

Concerning both vegetal and animal foodstuffs, these two methodologies are used in different applications [22,23,24]. Soxhlet is usually more diffuse in lab-scale and economical; on the other hand, supercritical CO_2_ is quite expensive but more compatible with food-grade and sustainable procedures.

The nitrogen compounds, peptides, and amino acids were obtained from the defatted products and the whole cheese by acidic extraction, centrifugation, and filtration. The use of HCl 0.1 M as extracting solvent allowed us to achieve the denaturation and precipitation of entire caseins, subsequently eliminated by the filtration step. Thus, the lower molecular weight (MW) nitrogen compounds (species generated by proteolytic processes and whey proteins) remain in solution.

To evaluate possible variations in the composition of the nitrogen fraction due to the defatting procedures, an amount corresponding to 1 g of starting material (untreated cheese, control sample) was used. For each extraction, 1.00, 0.65, and 0.66 g were used, respectively, to obtain the extracts from the whole cheese, and the samples defatted according to the different yields in defatted products.

### 3.2. Free Amino Acids in the PR Samples

**Figure 1:**
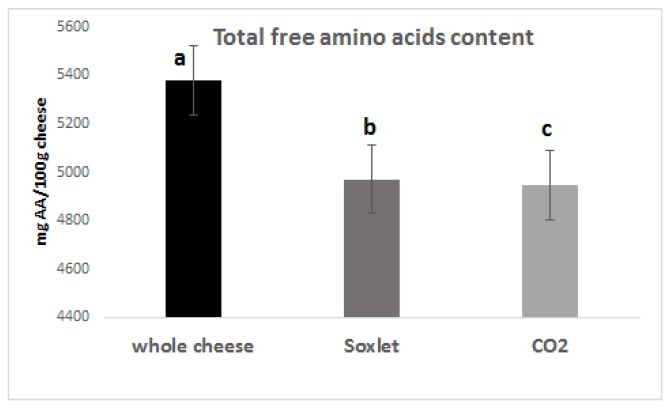
Total amount of free amino acids in the differently treated PR (mg amino acid/100 g PR; means ± SD). Bars carrying different letters are significantly different (*p* < 0.05) from each other.

Free amino acids account for up to 25% of the total nitrogen in PR [25], and they were reported to be, among the non-volatile nitrogen molecules, that ones mostly influence its taste [26,27]. Therefore, their preservation during the defatting process was considered as a critical factor for the comparison of the two methodologies used. The content of free amino acids was quantified in the extracts obtained by UPLC–ESI–MS analysis, after derivatization with the Waters AccQ-Fluor reagent kit, to enhance the chromatographic separation. Single ions were monitored for each amino acid (Table S1). The produced quantification results are reported in Figure 1.

The composition of the free amino acid fraction was found to be comparable with the data already reported by Careri et al. [28] for PR aged 24 months: with the most abundant amino acids being glutamic acid, lysine, proline, and leucine. However, little loss in the free amino acid content has been observed for both the defatted products, mainly due to the possible co-extraction of them with fat moiety. 

### 3.3. Characterization of Peptides and Proteins in the Aqueous Extracts

A UPLC–MS analysis of the acidic extracts was performed to evaluate their content in nitrogen compounds.

The mass spectra associated with the most intense chromatographic signals were analyzed to obtain the molecular masses of the most abundant nitrogen compounds. Non-proteolytic aminoacyl derivatives (NPADS), peptides, and proteins were identified starting from their deduced MW, by using the software “FindPept” tool [29] and “Proteomics Toolkit” [30] fragment ion calculator of the four bovine milk casein sequences from which the peptides originate. The in-source fragmentation signals were used to discriminate between possible identifications. 

The characterized compounds are reported in the supporting information (Table S1). The molecular weights of the found species were spread over a wide range; 12 lactoyl- and γ-glutamyl- derivatives of amino acids, already extensively characterized in cheese and other foods, and casein fragments of up to 81 amino acids were recognized. Moreover, α-lactalbumin and the isoforms A and B of β-lactoglobulin were detectable between 40 and 43 min.

Additionally, 19 of the 28 identified compounds have already been described by Sforza et al. (2012) [31] in a PR sample aged 24 months. It should be underlined that in the previous work, the species having an MW lower than 10 kDa were isolated and concentrated through an ultrafiltration process, resulting in a slightly different peptide mixture composition.

**Figure 2:**
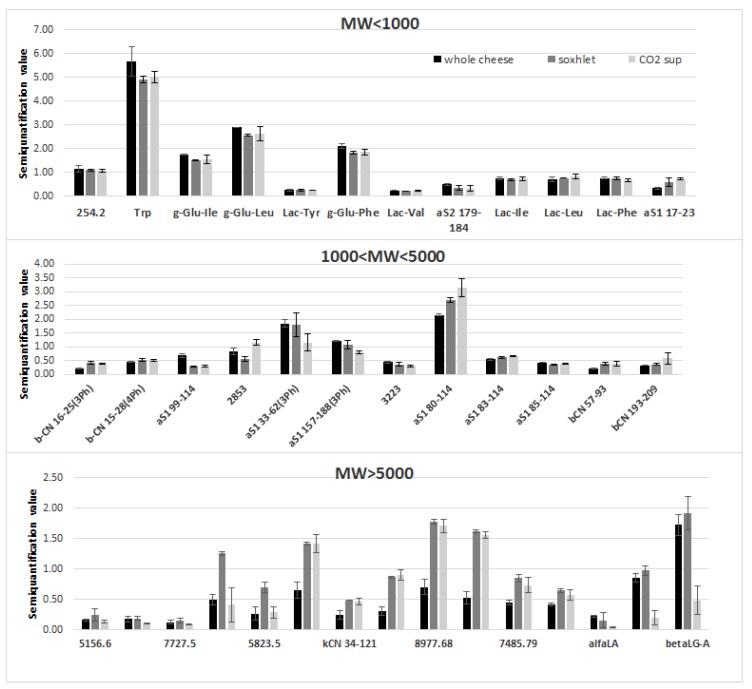
Semi-quantification of the main nitrogen compounds in the different PR extracts (means ± SD).

Each identified compound was then semi-quantified against the internal standard Phe-Phe, added during the extraction procedure, according to a method previously reported. [32] The obtained results are displayed in Figure 2.

The data presented in Figure 2 show that the whole cheese, Soxhlet, and supercritical CO_2_ samples seem to keep the same trend, except for the peptides group with MW >5000. In this case, higher amounts are observed for both the defatted samples compared with the control one. Maybe the presence of fat in the whole cheese prevented the extraction of high molecular weight peptides from the aqueous medium.

Altogether, data concerning the molecular characterization of the nitrogen fraction of the defatting products are comparable with the ones of the whole cheese, thus indicating that these new products keep the same nutritional features.

### 3.4. Simulated Gastrointestinal Digestion

Simulated gastrointestinal digestion, according to the procedure described by Minekus et al. [18] was performed on the defatted cheese samples and on the whole cheese, to detect if the digestibility of PR cheese is affected by defatting processes. As already done for the extraction, an amount corresponding to 1 g of starting material (untreated cheese, control sample) was used for each digestion. 

Free amino acids were quantified in the digests following the same procedure previously outlined for the extracts. 

**Figure 3:**
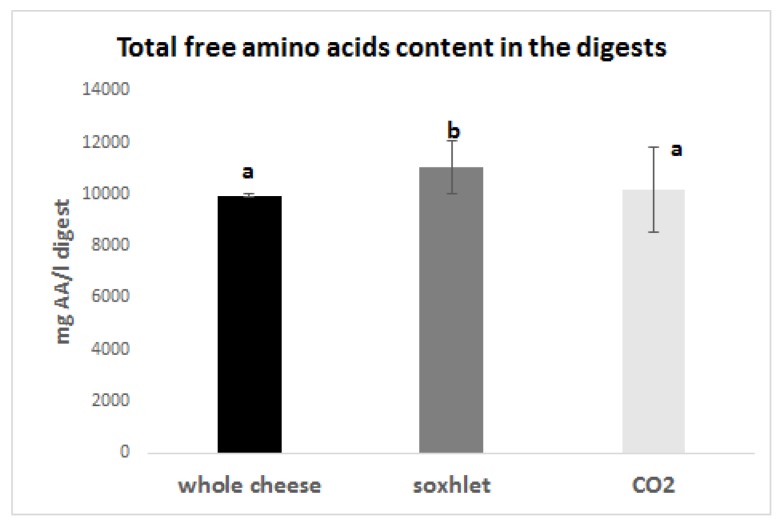
Total amount of free amino acids in the digested samples from differently treated PR (mg amino acid/100 g PR; means ± SD). Bars carrying different letters are significantly different (*p* < 0.05) from each other.

All the samples show an increase of free amino acid content after digestion, as compared with Figure 1. Interestingly, after digestion, the total free amino acid amount of the whole cheese was lower than that observed for the digested sample obtained from Soxhlet and quite similar to the one of supercritical CO_2_ extraction (Figure 3). This behavior suggests that the defatting processes could enhance the digestibility of PR: probably the lipids present in the whole cheese interfere with the digestive mixtures, lowering the efficiency of amino acid release. Defatted samples, instead, allow the peptides and proteins present to be more thoroughly digested. The ability of lipids present in milk to hamper the digestibility of proteins has already been reported in the literature. [33] 

Furthermore, the digestion products were characterized by LC–MS analysis to evaluate their content in peptides. This evaluation is particularly important since the digestion of dairy products has been reported to release many peptides exerting biological activities besides of their nutritional functions [34].

The most abundant nitrogen compounds were identified in the digested samples applying the same procedure outlined previously for the acidic extracts; the characterized species are reported in the supporting information (Table S2). Twenty-eight of the 64 identified compounds were already described by Bottari et al. (2017) [35] in the digests obtained from PR aged 16, 24, and 36 months.

Interestingly, none of the nitrogen compounds previously characterized in the acidic extracts were detected in significant amounts in the digested samples, except for NPADS. Compared with the species found in the acidic extracts, the molecular weight distribution was, as expected, considerably shifted towards lower values, with the most extended peptide being constituted by 23 amino acids.

### 3.5. Antioxidant Activity of the Digested Samples

**Figure 4:**
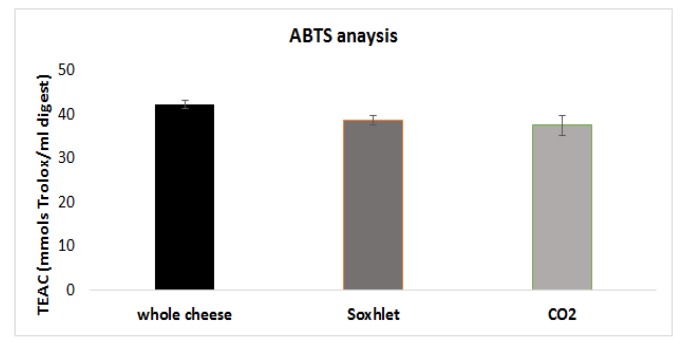
Antioxidant capacity (TEAC; mmols Trolox/mL digested sample) of the digested cheese samples. No significant differences are observed between the bars.

To evaluate the effect of the defatting process on the antioxidant properties of the PR samples after ingestion, the antioxidant capacity of the digested cheese samples was measured by a standard ABTS test [19]. The results were expressed in terms of Trolox equivalents (TEAC) related to the amount of digested PR (mmols of Trolox per digested sample in an amount corresponding to 100 g of the whole PR) and are reported in Figure 4.

As highlighted by the results reported in Figure 4, the data confirmed that, even after a digestion process, the biases introduced in the nitrogen fraction by the extraction procedure were still very visible. No statistically significant difference was found, although the antioxidant capacity of the defatted cheeses was slightly higher if compared to the one observed for the whole cheese.

From the detailed molecular composition of the nitrogen fraction determined, it is possible to speculate which species can be responsible for the scavenging activity observed. Among the total free amino acids, tyrosine is known to be the main one responsible for the antioxidant capacity of PR [36].

Known antioxidant descriptors for peptides include the presence of redox-active amino acids (Tyr, Trp, Met, Cys, and His) [37] and a limit on their length (the most active peptides are 3–10 amino acids long) [38]. Seven peptides identified fulfilled the first requirement.

Moreover, research in the BIOPEP [39] database also revealed peptides d13, d24, d27, and d43 to be included in (or completely covering) sequences known as antioxidants.

### 3.6. ACE inhibition Capacity of the Digested Samples

The angiotensin I-converting enzyme belongs to the renin–angiotensin system. It acts in the regulation of blood pressure, by cleaving the *C*-terminal dipeptide portion of angiotensin I and producing the vasoconstrictor angiotensin II. Some peptides, which possess a specific *C*-terminal sequence, can bind to ACE, and they represent competitive substrates for the enzyme mentioned above. Peptides released during proteolytic processes in food, such as cheese ripening, share these features and, therefore, they have been studied and assessed for their in vitro ACE-inhibition potential [40,41].

**Figure 5:**
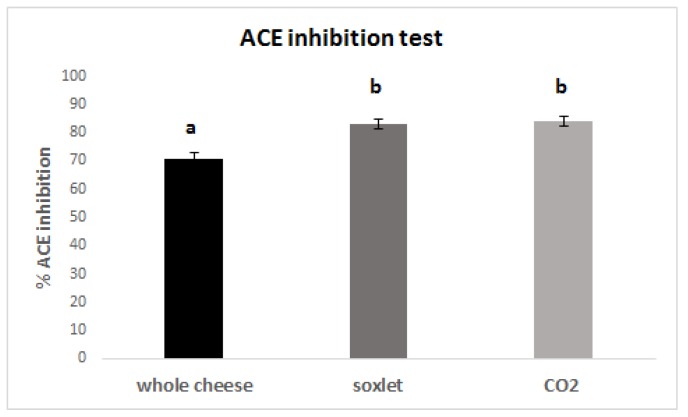
Percentages of ACE inhibition of the digested cheese samples. Bars carrying different letters are significantly different (*p* < 0.05) from each other.

To also evaluate the influence of the defatting processes on the presence of ACE inhibiting compounds in the digested samples, the ACE inhibition capacity was measured according to a procedure reported in the literature [20,21]. The results were expressed as percentages of ACE inhibition and are reported in Figure 5.

Again, the effect of the defatting procedures was very evident also after the digestion. The samples treated with Soxhlet and supercritical CO_2_ show the most intense ACE inhibiting activity. This is likely a consequence of the selective enrichment of the fraction in short peptides, which are known to be the most effective, generally speaking, in ACE inhibition.

Exploring the peptide sequences identified in the digested samples (Table S2), it was found that some residues (MPFPK from peptide d36, FVAP from peptide d46, and YPFGPIPN from peptide d55) were already found to be part of potential ACE inhibitory peptides in Cheddar and Gouda cheeses [40,41].

It should be noted that most of the studies reported in literature directly investigated the ACE inhibitory activity of peptides in aqueous extracts of cheeses and not of peptides generated by their gastrointestinal digestion. 

Further research in the BIOPEP database [35] also revealed peptides d4, d13, d24, d36, d46, and d55 to be included in (or completely covering) sequences known as ACE inhibitors.

## 4. Conclusions

Based on the total protein content, free amino acids, and other nitrogen compounds (NPADs, peptides, and proteins) identified in the acidic extracts and in the gastrointestinal digestion products, it is evident how Soxhlet and supercritical CO_2_ extraction can be applied to obtain defatted products from PR cheese since they allow the minimization of the loss of nitrogen compounds. Moreover, the tested samples also revealed that the defatting procedures seem to enhance the digestibility of PR. More studies will also be performed to test other PR cheeses with different aging, and the consumer attitudes towards flavor, texture, and general acceptance of the defatting product would also be considered as part of future studies.

Testing some bio-functionalities after simulated digestion indicated that the biases introduced by the defatting procedures remain evident also in the biological activity, even if the digestion itself completely changes the composition of the peptide fraction. The measurement of bio-functional properties of the digests highlighted that Soxhlet and supercritical CO_2_ extraction allowed us to obtain products with antioxidant capacities, which were comparable to the ones of whole cheese. At the same time, the ACE inhibitory activities were found to be even enhanced by these two defatting procedures. The measured activities were related to the nitrogen species contained in the digested samples. More studies will be performed to better elucidate and identify the peptide sequences involved in these bioactive mechanisms. 

## Figures and Tables

**Table 1:** Total nitrogen and fat determination.

Sample	Protein (%)	Fat (%)
Whole Cheese	35.7 ± 0.1	37.7 ± 0.4
Soxhlet-defatted	50.7 ± 2.2	2.6 ± 0.2
CO_2_-defatted	46.2 ± 0.9	4.6 ± 0.1

## Supplementary Materials

The following are available online at https://www.mdpi.com/2304-8158/9/3/310/s1. Table S1: Characteristic ions, chromatographic retention time and potential identification for the main nitrogen compounds found in the PR extracts; Table S2: Characteristic ions, chromatographic retention time and potential identification for the main nitrogen compounds found in the PR extracts in PR digests.
