# Supplementary material for: Characterization of Defatted Products Obtained from the Parmigiano–Reggiano Manufacturing Chain: Determination of Peptides and Amino Acids Content and Study of the Digestibility and Bioactive Properties
Source: Foods. 2020 Mar 9;9(3):310. doi: 10.3390/foods9030310 (PMC7143180; doi:10.3390/foods9030310)
Supplement: Supplementary file 1 [file foods-09-00310-s001.zip › foods-693334-supplementary.docx]

**Supporting information.**

**Table S1.** Characteristic ions, chromatographic retention time and potential identification for the main nitrogen compounds found in the PR extracts.

|  | MW | Ions | Retention time (min) | Identification |
| --- | --- | --- | --- | --- |
| p1 | 189 | 190.0 + 172.3 | 17.39 | Lac-Val |
| p2 | 203 | 204.1 + 158.0 | 15.06 | Lac-Ile |
| p3 | 203 | 204.1 + 158.0 | 15.67 | Lac-Leu |
| p4 | 205 | 205.1 + 188.1 | 14.42 | Trp |
| p5 | 237 | 238.1 + 166.0 | 23.65 | Lac-Phe |
| p6 | 253 | 254.2 | 11.62 | Lac-Tyr (diastereomer) |
| p7 | 253 | 254.2 | 16.32 | Lac-Tyr  (diastereomer) |
| p8 | 260 | 261.2 + 132.0 | 21.71 | γ-Glu-Ile |
| p9 | 260 | 261.2 + 132.0 | 21.92 | γ -Glu-Leu |
| p10 | 294 | 295.1 + 166.0 | 17.03 | γ -Glu-Phe |
| p11 | 786 | 786.5 + 393.8 | 21.03 | α-S2 179-184 |
| p12 | 904 | 905.6 + 453.4 | 27.84 | α-S1 17-23 |
| p13 | 1347 | 1348.4 + 674.9 | 18.39 | β-CN 16-25(3Ph) |
| p14 | 1870 | 936.0 + 624.3 | 22.48 | β-CN 15-28(4Ph) |
| p15 | 1880 | 941.3 + 627.8 | 35.95 | β-CN 193-209 |
| p16 | 1937 | 969.9 + 646.9 + 485.5 | 25.7 | α-S1 99-114 |
| p17 | 2853 | 952.1 + 714.3 + 571.6 + 476.6 | 26.42 | - |
| p18 | 3223 | 806.5 + 645.7 + 538.1 | 27.91 | - |
| p19 | 3452 | 864.1 + 691.4 + 576.3 + 494.1 | 27.7 | α-S1 157-188(3Ph) |
| p20 | 3580 | 1194.6 + 896.0 + 717.1 + 597.7 + 512.4 | 27.07 | α-S1 33-62(3Ph) |
| p21 | 3602 | 901.6 + 721.4 | 34.48 | α-S1 85-114 |
| p22 | 3859 | 965.9 + 772.8 + 644.2 + 549.8 | 33.18 | α-S1 83-114 |
| p23 | 4024 | 1342.3 + 1006.9 | 41.51 | β-CN 57-93 |
| p24 | 4238 | 1060.2 + 848.5 + 707.3 + 606.3 | 32.67 | α-S1 80-114 |
| p25 | 5029 | 1007.0 + 839.1 + 719.3 | 28.59 | - |
| p26 | 5156 | 1032.4 + 860.4 + 737.6 + 645.6 | 28.11 | - |
| p27 | 5823 | 833.0 + 728.8 + 647.8 + 583.1 | 29.98 | - |
| p28 | 5950 | 992.9 + 851.0 + 744.7 + 662.1 + 596.1 | 29.72 | - |
| p29 | 7490 | 1499.0 + 1249.5 + 937.4 | 38.81 | - |
| p30 | 7727 | 1288.7 + 1104.7 + 967.3 + 859.6 | 28.64 | - |
| p31 | 7734 | 1547.8 + 1289.9 + 967.7 | 38.83 | - |
| p32 | 8716 | 1453.9 + 1246.1 + 1090.6 + 969.4 + 872.7 + 793.5 + 727.3 | 37.22 | β-CN 102-178 |
| p33 | 8756 | 1460.8 + 1251.9 + 1095.5 + 973.8 + 876.7 | 35.97 | - |
| p34 | 8895 | 1271.8 + 1113.0 + 989.1 + 890.3 + 809.6 + 742.3 | 35.27 | k-CN 34-121 |
| p35 | 8982 | 1497.9 + 1284.2 + 1123.6 + 998.8 + 899.0 + 817.6 + 749.2 | 36.11 | - |
| p36 | 9023 | 1504.7 + 1290.0 + 1128.8 + 1003.5 + 903.2 + 821.2 + 752.7 + 695.1 | 34.89 | β-CN 64-144 |
| p37 | 14181 | 1774.0 + 1577.0 + 1418.8 + 1290.0 + 1182.6 + 1092.1 | 40.95 | α-LA |
| p38 | 18280 | 1407.2 + 1306.5 + 1219.6 + 1143.6 + 1076.1 + 1617.2 | 41.55 | β-LG-B |
| p39 | 18367 | 1413.7 + 1312.8 + 1225.3 + 1148.8 + 1081.3 + 1021.3 | 42.03 | β-LG-A |

**Table S2.** Characteristic ions, chromatographic retention time and potential identification for the main nitrogen compounds found in the digested PR samples; species found also in the acidic extracts are coded by the letter **p**, while the molecules formed by the digestion process are identified by the letter **d**.

|  | MW | Ions | Retention time (min) | Identification |
| --- | --- | --- | --- | --- |
| p1 | 189 | 190.0 + 172.3 | 17.5 | Lac-Val |
| p2 | 203 | 204.1 + 158.0 | 21.73 | Lac-Ile |
| p3 | 203 | 204.1 + 158.0 | 21.94 | Lac-Leu |
| d1 | 230 | 231.3 | 13.49 | VI/IV/VL/LV |
| p5 | 237 | 238.1 + 166.0 | 23.66 | Lac-Phe |
| p6 | 253 | 254.2 | 11.82 | - |
| p7 | 253 | 254.2 | 16.31 | Lac-Tyr |
| p8 | 260 | 261.2 + 132.0 | 15.11 | γ-Glu -Ile |
| p9 | 260 | 261.2 + 132.0 | 15.69 | γ-Glu -Leu |
| d2 | 294 | 295.3 + 136.1(a1) | 17.95 | YI/ YL |
| p10 | 294 | 295.1 + 166.0 | 17.06 | γ-Glu -Phe |
| d3 | 317 | 318.3 + 300.3(b3) + 213.3(b2) + 185.3(a2) | 12.9 | β-CN 162-164 |
| d4 | 345 | 346.3 + 328.3(b3) | 15.16 | α-S1 129-131 |
| d5 | 375 | 376.3 | 25.43 | - |
| d6 | 457 | 458.3 + 230.2(y2) | 26.2 | α-S1 181-184 |
| d7 | 469 | 470.4 | 18.8 | - |
| d8 | 474 | 475.4 + 229.2(y2) | 22.57 | β-CN 184-187 |
| d9 | 490 | 491.4 + 229.2(b2) | 23.38 | β-CN 116-119 |
| d10 | 520 | 521.4 | 11.79 | - |
| d11 | 525 | 526.4 + 227.2(y2) | 15.16 | α-S1 135-139 |
| d12 | 529 | 530.4 + 229.3(y2) | 28.34 | β-CN 218-222 |
| d13 | 548 | 549.4 + 275.2 + 136.1(a1) + 235.2(b2) | 13.28 | k-CN 61-65 |
| d14 | 552 | 553.4 | 20.47 | - |
| d15 | 561 | 562.4 + 360.2(y3) | 23.43 | α-S1 194-198 |
| d16 | 570 | 571.4 | 18.53 | - |
| d17 | 587 | 588.4 | 15.28 | - |
| d18 | 587 | 588.5 + 263.3(y2) | 31.92 | β-CN 115-119 |
| d19 | 588 | 589.4 | 19.97 | - |
| d20 | 603 | 604.4 | 20.47 | - |
| d21 | 627 | 628.5 | 24.11 | - |
| d22 | 644 | 645.4 + 528.2(b5) | 25 | α-S1 143-148 |
| d23 | 645 | 646.4 | 13.69 | - |
| d24 | 652 | 652.5 + 440.2(y4) + 244.3(y2) | 22.33 | β-CN 170-175 |
| d25 | 653 | 654.4 + 229.2(y2) | 13.66 | α-S1 165-169 |
| d26 | 667 | 668.5 | 25 | - |
| d27 | 673 | 674.4 + 504.3(y4) | 19.37 | β-CN 177-182 |
| d28 | 673 | 674.4 | 21.97 | - |
| d29 | 674 | 675.4 | 16.1 | - |
| d30 | 676 | 677.4 + 362.2(y3) | 19.13 | β-CN 126-131 |
| d31 | 683 | 684.5 | 24.05 | - |
| d32 | 688 | 689.5 + 461.4(b4) | 28.84 | β-CN 133-138 |
| d33 | 705 | 706.4 + 353.7 + 504.4(y4) | 16.72 | α-S1 53-58 |
| d34 | 730 | 731.5 | 17.99 | - |
| d35 | 741 | 742.6 + 625.6(b6) + 512.4(b5) | 33.9 | β-CN 203-209 |
| d36 | 748 | 748.5 + 488.4(y4) + 244.3(y2) | 22.06 | β-CN 108-113 |
| d37 | 750 | 751.5 + 586.5(b5) + 489.3(b4) | 26.99 | β-CN 114-119 |
| d38 | 754 | 755.6 + 668.5(y6) + 440.3(y4) | 24.92 | α-S1 180-186 |
| d39 | 757 | 758.6 + 344.3(y3) | 28.08 | α-S1 25-31 |
| d40 | 781 | 782.5 + 391.8 + 636.3(b6) | 14.1 | α-S1 135-141 |
| d41 | 792 | 793.5 | 19.97 | - |
| d42 | 812 | 812.6 + 596.4(b6) | 21.2 | α-S2 115-122 |
| d43 | 821 | 821.6 + 609.3(y5) | 28.17 | β-CN 162-168 |
| d44 | 838 | 838.6 | 25.55 | - |
| d45 | 888 | 889.6 + 627.5(y6) | 31.85 | β-CN 59-66 |
| d46 | 904 | 905.7 + 588.5(y5) + 344.3(y3) | 31.99 | α-S1 24-31 |
| d47 | 907 | 907.6 | 20.47 | - |
| d48 | 907 | 907.7 | 26.2 | - |
| d49 | 910 | 910.7 + 455.9 | 12.66 | α-S1 135-142 |
| d50 | 985 | 985.6 | 17.95 | - |
| d51 | 1001 | 1001.8 | 27.08 | - |
| d52 | 1008 | 1008.8 | 27.57 | - |
| d53 | 1013 | 1013.7 + 507.3 + 338.8 | 18.28 | β-CN 106-113 |
| d54 | 1096 | 1096.7 | 19.89 | - |
| d55 | 1100 | 1100.8 + 550.9 + 871.7(b8) | 29.83 | β-CN 59-68 |
| d56 | 1124 | 1124.8 + 563.0 | 32.06 | - |
| d57 | 1136 | 1136.9 + 568.9 | 26.2 | - |
| d58 | 1221 | 1221.8 + 611.5 + 977.8(b9) | 33.32 | β-CN 81-91 |
| d59 | 1259 | 1259.9 + 630.4 + 1031.8(b9) | 34.95 | β-CN 129-139 |
| d60 | 1282 | 1282.9 + 641.9 | 18.5 | - |
| d61 | 1300 | 1301.0 + 651.0 + 1071.8(b10) + 838.6(y8) | 32.45 | β-CN 57-68 |
| d62 | 1373 | 1373.9 + 687.5 + 1145.8(b11) + 934.6(b9) | 30.48 | α-S1 174-186 |
| d63 | 1574 | 1576.1 + 788.1 | 36.23 | β-LG 31-44 |
| d64 | 2512 | 1256.9 + 838.4 + 653.5(b6) | 36.83 | β-CN 69-91 |
